# Calcifying Epithelial Odontogenic Tumor in Anterior Maxilla Associated with a Supernumerary Tooth: A Case Report

**DOI:** 10.5681/joddd.2013.009

**Published:** 2013-02-21

**Authors:** Urvashi Sharma, Anubha Gulati, Hemant Batra, Devinderpreet Singh

**Affiliations:** ^1^Assistant Professor, Department of Pedodontics, Dr. HSJ Institute of Dental Sciences and Hospital, Panjab University, Sector 25, Chandigarh, India; ^2^Associate Professor, Department of Oral Pathology, Dr. HSJ Institute of Dental Sciences and Hospital, Panjab University, Sector 25, Chandigarh, India; ^3^Professor, Department of Oral & Maxillofacial Surgery, Dr. HSJ Institute of Dental Sciences and Hospital, Panjab University, Sector 25, Chandigarh, India; ^4^Assistant Professor, Department of Orthodontics, Dr. HSJ Institute of Dental Sciences and Hospital, Panjab University, Sector 25, Chandigarh, India

**Keywords:** Calcifying epithelial odontogenic tumor, intraosseous, maxilla, odontogenic tumors, supernumerary tooth

## Abstract

Odontogenic tumors are derived from epithelial, ectomesenchymal and/or mesenchymal elements that are or have been a part of the tooth-forming apparatus. Of all the odontogenic tumors, calcifying epithelial odontogenic tumor accounts for 1% of the cases. Approximately 200 cases have been reported to date. There is no sex predilection, with a 2:1 predilection for the mandible, mostly in the premolar/molar region. It is often locally invasive. Most often, it is associated with an impacted tooth, is asymptomatic and requires biopsy for diagnosis. Presented here is a rare case of an intraosseous calcifying epithelial odontogenic tumor surrounding a supernumerary tooth. Furthermore, the occurrence of this tumor in the anterior maxilla (an uncommon site) in a pediatric patient makes it rarer. Although the present case was asymptomatic, root resorption and displacement of adjacent teeth necessitated its surgical removal. The lesion was surgically enucleated and histopathological examination confirmed calcifying epithelial odontogenic tumor, showing abundant calcifications in the form of Liesegang rings.

## Introduction

Calcifying epithelial odontogenic tumor is an asymptomatic, benign, slow-expanding and a locally invasive tumor. Studies reveal an approximate 1% prevalence rate of this tumor amongst all odontogenic tumours.^1^-^3^ Different terminologies have been used for this tumor, such as ameloblastoma of unusual type with calcification, calcifying ameloblastoma, malignant odontoma and cystic complex odontoma.^2^

The peak occurrence of this tumor is at 40 years of age although an age range of 8-92 years has been reported.^4^ It may be classified as intraosseous or extraosseous. The extraosseous variant has a predilection for anterior gingiva where it appears as a sessile mass capable of destroying the underlying bone. Intraosseous type is more commonly found in the mandible and more so, in the posterior region. More than half of these are associated with an impacted tooth.^5^ The occurrence of a supernumerary tooth in the present case is in itself a rarity.^6^ The differential diagnosis includes adenomatoid odontogenic tumor, calcifying odontogenic cyst, dentigerous cyst, ameloblastic fibro-odontoma and odontoma.^2,7^

The present case reports a rare case of an intraosseous calcifying epithelial odontogenic tumor in the anterior maxilla (an uncommon site), causing root resorption and displacement of adjacent teeth. The occurrence in a pediatric patient, aged 13, is still rare. However, numerous calcifications were evident histologically, indicating a much earlier age of onset of this tumor.

## Case report

A 13-year-old boy referred to the Department of Pedodontics with a chief complaint of irregular teeth. The patient had been referred by an orthodontist who had placed orthodontic brackets on selected incisors as well as primary maxillary right canine. Radiographic examination had been ignored prior to treatment, leading to erroneous placement of a bracket on the primary canine.

An extra-oral examination showed no swelling or lymphadenopathy. The intra-oral examination showed a complete set of permanent teeth in the left maxillary area up to the second molars but on the right side, the primary canine was firmly retained. However, its permanent counterpart was not visible intra-orally. There was neither a canine bulge nor a cortical expansion of the maxilla. The mandibular arch had a full complement of permanent teeth up to the first molars except the mandibular second primary molars which were near exfoliation.

**Figure 1:**
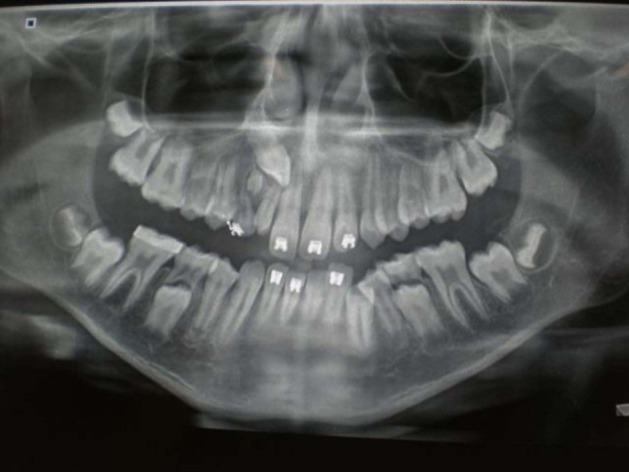
Panoramic revealed a supernumerary tooth in a cystic space, causing divergence of 12 and 53.

An orthopantomograph revealed a supernumerary tooth-like structure between the roots of permanent maxillary right lateral incisor and primary maxillary right canine. It was enclosed in a unilocular cystic space, which had caused divergence of the roots of these teeth (Figure 1). In addition, there was an overlying impacted, mesially rotated permanent right canine. Radiologically, the lesion resembled a compound odontoma or a dentigerous cyst.

**Figure 2:**
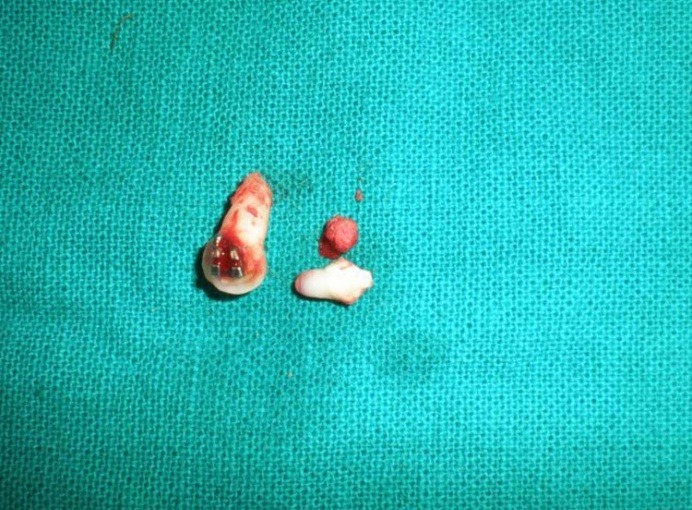
The extracted primary canine and the supernumerary tooth with the cystic tissue.

The over-retained primary canine was extracted. The embedded tooth-like structure was surgically enucleated along with its cyst-like lining (Figure 2). It had a conical crown and a short root with a closed apex. It was then sent for histopathological examination. The patient is currently undergoing comprehensive fixed mechanotherapy to facilitate eruption of impacted maxillary right canine.

**Figure 3:**
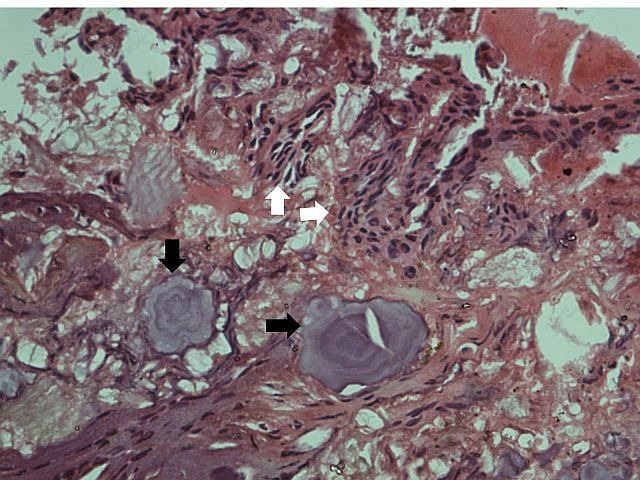
Photomicrograph showing Liesegang rings (black arrows) and strands of polyhedral epithelial cells (white arrows) with pleomorphic nuclei (H & E ×400)

Histopathological examination showed strands of polyhedral epithelial cells with pleomorphic nuclei and calcification in the form of Liesegang rings (Figure 3). The diagnosis confirmed calcifying epithelial odontogenic tumor. On Congo red staining, the tissue specimens showed mainly blood and fibrin with few calcified areas and scant epithelial rests. The Congo red staining was negative for amyloid.

## Discussion

Calcifying epithelial odontogenic tumor is a benign epithelial odontogenic tumor and is also termed Pindborg tumor after the name of the founder in 1955.^8^ Most cases (94%) are intraosseous and only 6% are extraosseous.^2^ The origin of this tumor is uncertain. Some authors speculate that it originates from the remnants of dental lamina based on its anatomic distribution in the jaws and/or basal cells of gingival surface epithelium.^2^ Still others suggest that it bears structural resemblance to the cells of stratum intermedium of the enamel organ along with a high activity of alkaline phosphatase and adenosine triphosphate.^4^

Radiographically, the intraosseous lesion presents as radiolucency. Later, as the lesion ages, calcium salts are deposited and it becomes increasingly radio-opaque. It also simultaneously erodes bone and thus, the lesion is often mixed radiolucent/radio-opaque giving a characteristic ‘driven snow’ appearance on the radiograph. Further, the lesion may be unilocular or more commonly, multilocular in appearance. Sixty percent of intraosseous variants often involve one or more impacted teeth.^6^ Kaplan et al reported 41 cases of one or more impacted teeth (60%) associated with a total of 67 cases of calcifying epithelial odontogenic tumor.^6^ Out of these, the most prevalent were the molars (62%) followed by premolars, canines, incisors and the least were the supernumerary or unidentified teeth (4%). In our case, the radiolucency was pericoronal and unilocular with a thin sclerotic border, containing a supernumerary tooth bearing resemblance to a dentigerous cyst.

Pathological reports of calcifying epithelial odontogenic tumor exhibit considerable variations. It is characterized by a fibrous tissue stroma with sheets or islands of polyhedral epithelial cells with intercellular bridges. Nuclei are often pleomorphic and giant nuclei may be visible. Eosinophilic, amorphous hyaline-rich material, which stains positive for amyloid, may be present. Calcifications in the form of concentric rings, called Liesegang rings, may develop within the amyloid-like material. This material stains with Congo red and exhibits an apple-green birefringence under polarized light. It also fluoresces under ultraviolet light with thioflavin T. This amyloid-like material may contain either basement membrane components (type IV collagen)^9^ or a mixture of cytokeratins.^10^ The origin of amyloid is unclear. It could either be an active secretion product or a degeneration product of keratin filaments which originate from tumor epithelium due to developmental or aging processes.^10^

Some of these tumors may be epithelium-predominant with minimal amyloid whereas others may be amyloid-predominant with small islands of epithelium. Still others may have abundant clear cells.^11^ A mixed lesion along with adenomatoid odontogenic tumor has also been reported.^12^ The given section in our case revealed islands and strands of polyhedral epithelial cells in a fibrous stroma. The fibrous stroma revealed the presence of numerous calcifications, suggestive of lesion progression and a lesion of long standing. Some of these were in the form of concentric rings, also called Liesegang rings. Congo red testing for amyloid was negative in the present case as the amyloid had become calcified.

Calcifying epithelial odontogenic tumor is less aggressive than ameloblastoma although cases of malignant transformation have been reported.^13^ The aggressiveness is a prominent finding in posterior maxilla. In addition, root resorption is reported as a rare finding in calcifying epithelial odontogenic tumor (4%), unlike solid ameloblastoma (81%).^6^ However, in the present case, root resorption of the primary canine was evident, which could be secondary to exfoliation or pressure exerted by the tumor itself. The latter was more likely as the roots of primary canine and permanent lateral incisor were laterally displaced by the growing tumor.

Displacement of adjacent teeth was also observed in the present case. Growth of the tumor coupled with local invasiveness might be possible etiologic factors. Kaplan et al reported 28 cases out of a total of 67 cases (41%) of calcifying epithelial odontogenic tumor which had caused displacement of teeth.^6^

Treatment of calcifying epithelial odontogenic tumor involves enucleation of smaller lesions and resection of large ones.^5^ The resection should include a rim of the surrounding bone. A long follow-up is recommended as a recurrence rate of 14% has been observed, particularly for those which have been curetted.^14^ The lesion in the presented case was enucleated after the primary canine was extracted. A one-year follow-up revealed uneventful healing and no recurrence was noticed. Literature search, however, arbitrarily states a minimal follow-up of five years.^2,4^ The patient is therefore on frequent recall visits.

The intraosseous variant of calcifying epithelial odontogenic tumor in the anterior maxilla and its association with a supernumerary tooth is in itself a rare entity coupled with its occurrence in a pediatric patient. In the present case, the patient was totally asymptomatic and hence, the correlation of clinical, radiographic and histopathologic examination is stressed upon, particularly if the contralateral tooth has not erupted for a considerable period.
